# Nanofabrication of synthetic nanoporous geomaterials: from nanoscale-resolution 3D imaging to nano-3D-printed digital (shale) rock

**DOI:** 10.1038/s41598-020-78467-z

**Published:** 2020-12-09

**Authors:** Jan Goral, Milind Deo

**Affiliations:** Department of Chemical Engineering, University of Utah, Salt Lake City, UT USA

**Keywords:** Petrology, Characterization and analytical techniques, Imaging techniques, Microscopy, Nanofluidics, Nanopores, Structural properties

## Abstract

Advances in imaging have made it possible to view nanometer and sub-nanometer structures that are either synthesized or that occur naturally. It is believed that fluid dynamic and thermodynamic behavior differ significantly at these scales from the bulk. From a materials perspective, it is important to be able to create complex structures at the nanometer scale, reproducibly, so that the fluid behavior may be studied. New advances in nanoscale-resolution 3D-printing offer opportunities to achieve this goal. In particular, additive manufacturing with two-photon polymerization allows creation of intricate structures. Using this technology, a creation of the first nano-3D-printed digital (shale) rock is reported. In this paper, focused ion beam-scanning electron microscopy (FIB-SEM) nano-tomography image dataset was used to reconstruct a high-resolution digital rock 3D model of a Marcellus Shale rock sample. Porosity of this 3D model has been characterized and its connected/effective pore system has been extracted and nano-3D-printed. The workflow of creating this novel nano-3D-printed digital rock 3D model is described in this paper.

## Introduction

Materials are synthesized to achieve a certain engineered characteristic. Micro- and nanofabricated structures are used in separations, chromatography, sensing, and many more applications. Study of the synthesized structures may be looked at from two vantage points. The first, which is intuitive and direct, is where a porous material is created to achieve a certain design specification in separation or sensing. From this perspective, it is important to create materials that are homogeneous, uniform, and repeatable, assuming that these characteristics help achieve the specific objective the material is being designed for. In recent years, a slightly different perspective has emerged, which is to recreate natural porous materials in order to understand the fluid behavior inside their pores. Naturally-occurring materials tend to be complex, variable, and heterogeneous. It is also not possible to easily find samples that have similar enough features, so that their behavior may be tested reproducibly. Another motivation for these examinations arises from an understanding that fluid flow and fluid thermodynamic behavior inside the pores of diameters of the order of few nanometers are significantly different from bulk phenomena^1,2^. In a recent paper, Xia et al.^3^ showed that the freezing behavior of water deviated significantly from the bulk in materials characterized by sub-10 nm pores. In order to study complex porous materials from this second vantage point (understanding fluid behavior), the nature of the porous materials must be known. Advanced imaging and image analysis techniques have made it possible to image both the manmade and natural materials in 3D at ultra-high resolution. The next step is to be able to recreate the imaged content reproducibly. Shales may benefit from this type of scrutiny because of their dominant contribution to the domestic crude oil and natural gas production in the United States.

Crude oil production in the United States in the first quarter of 2020 was on average 12.7 million barrels of oil a day, more than half of which was from hydraulically-fractured tight-oil rock formations—unconventional (shale) reservoirs commonly referred to as shales^4,5^. The global COVID-19 pandemic and low resultant demand have brought the production down. Nevertheless, it is crucial to understand the nanoporous structure of shales to improve recoveries from these important resources. Numerous studies have shown that shales are complex and inherently heterogeneous across multiple length scales, and are characterized by nanometer-sized pores^6–13^. The morphological and compositional heterogeneity of geological materials, which results in complex multi-scale fluid flow and transport phenomena, motivates the need for developing novel geo-architected materials suited for more sophisticated experiments to validate fluid flow models recently developed by numerus research groups^14–17^. There is considerable evidence that the known laws of adsorption, reaction, phase transitions, and flow behavior are affected by the presence of fluids confined in porous materials with nanometer-sized pores^18,19^. For the understanding of these behaviors, and for the creation of new laws that govern physical processes in nano/micro pore systems, making perfectly calibrated (repeatable) materials that mimic the pore structure of shales is necessary.

3D-printing is becoming more common across a large number of applications. A comprehensive review of emerging applications in this field is outside the scope of this document. Numerous examples are found in additive manufacturing, biomedical devices, tissue engineering, and composite manufacturing^20–27^. Advantages of 3D-printing include perfectly repeatable object creation, and possibly benefits of cost and speed of manufacturing. Rapid prototyping of complex geo-objects makes it possible to test important processes and phenomena. Ishutov et al.^28^ reviewed the application of 3D-printing in creating repeatable geomaterials. With all the advances in 3D-printing, developing a robust printing protocol at the nanoscale-resolution has been challenging. Xiong et al.^29^ had shown that a combination of additive (two-photon polymerization) and subtractive (laser ablation) technologies could be used for making highly reproducible intricate microstructures. Salmean and Dimartino^30^ describe 3D-printing of stationary phases with ordered morphology using additive manufacturing technology. They note that in the area of chromatography, 3D-printing afforded the ability to fabricate precisely ordered structures providing significant improvement over the conventional slurry packing methods. 3D-printing provided an avenue to achieve stationary phase homogeneity. While homogeneity was one of the main characteristics highlighted in this review, several other features of 3D-printing for customizing columns for specific applications were also pointed out. For the application involving 3D-printing complex natural features, the reproducibility of the specificity of the features is attractive. The two-photon polymerization process for 3D-printing at this resolution has been discussed in a few other applications^31–35^. Thus, the possibility of being able to ‘print’ a 3D structure of nanoporous geomaterials has been evident. However, there are a number of challenges of reconstructing realistic (representative) synthetic rocks and of actually being able to print and validate these 3D models. Some of these challenges include, but are not limited to: collection a high-quality 3D image dataset of a non-conductive materials (such as shale rocks) at nanoscale-resolution; 3D image processing, segmentation, and reconstruction using advanced image analysis tools; identification of pores that are responsible for hydrocarbon production using pore network modeling; and finally how to fabricate these pore systems for fluid flow experiments. These challenges were successfully addressed in this work, and for the first-time a digital rock 3D model was nano-3D-printed. The workflow and results are discussed in the paper.

The first step in creating representative synthetic rocks is to extract information from geological samples at very high resolution. It has been shown in multiple previous studies on shale 3D-imaging/-modeling, that attaining nanoscale-resolution is crucial in being able to replicate fluid behavior^36–42^. Thus, reconstructing digital rock 3D models from high-resolution image datasets is an important step in the workflow described in this paper. This was accomplished in this work using nanoscale-resolution focused ion beam scanning electron microscopy (FIB-SEM) nano-tomography (serial-sectioning)^43,44^. FIB-SEM serial-sectioning, and/or other 3D imaging techniques (such as micro/nano X-ray computed tomography (micro-/nano-CT)), are becoming more popular in the petroleum industry’s petrophysical labs to image shales in 3D and replicate their nanoporous structure into digital rock 3D models of various phases^45^. The pore phase of these 3D models can be then characterized and separated into connected (effective) porosity and non-connected (isolated) porosity using pore network modeling (PNM) method. The effective pore system—pores responsible for fluid flow, and hence permeability of shales—can be then extracted and nano-3D-printed for microfluidics (lab-on-a-chip) experiment purposes.

## Results and discussion

### Pore network modeling

**Figure 1:**
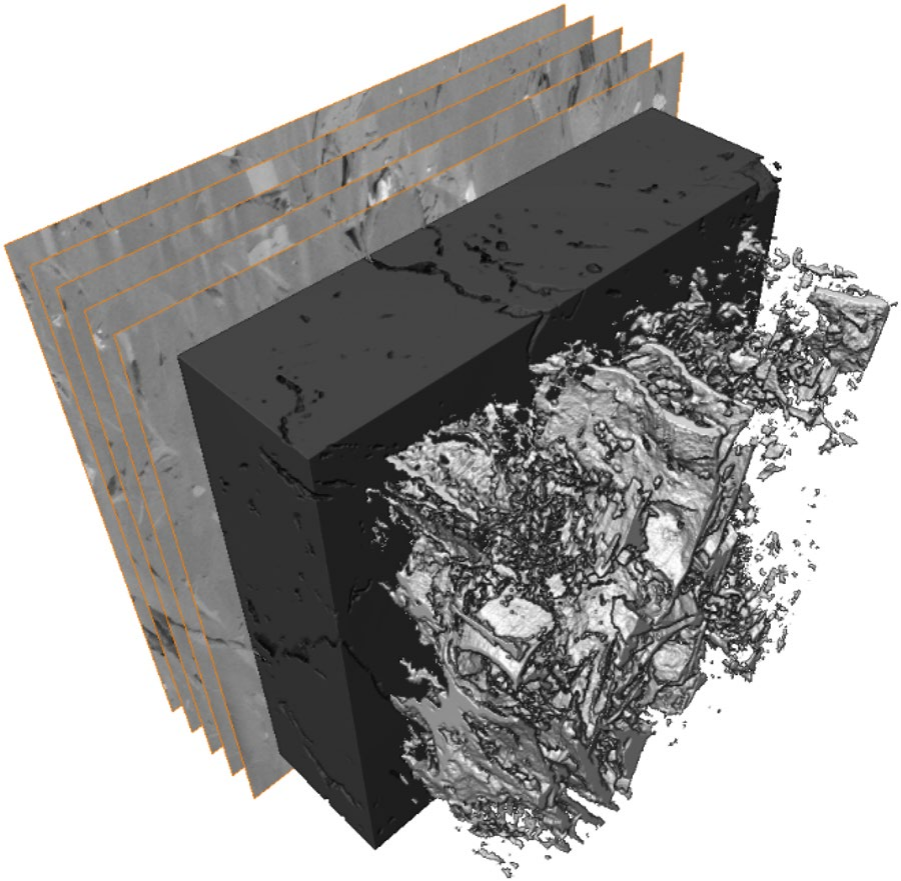
Visualization of the digital rock 3D model reconstructed from processed and segmented FIB-SEM nano-tomography image dataset.

**Figure 2:**
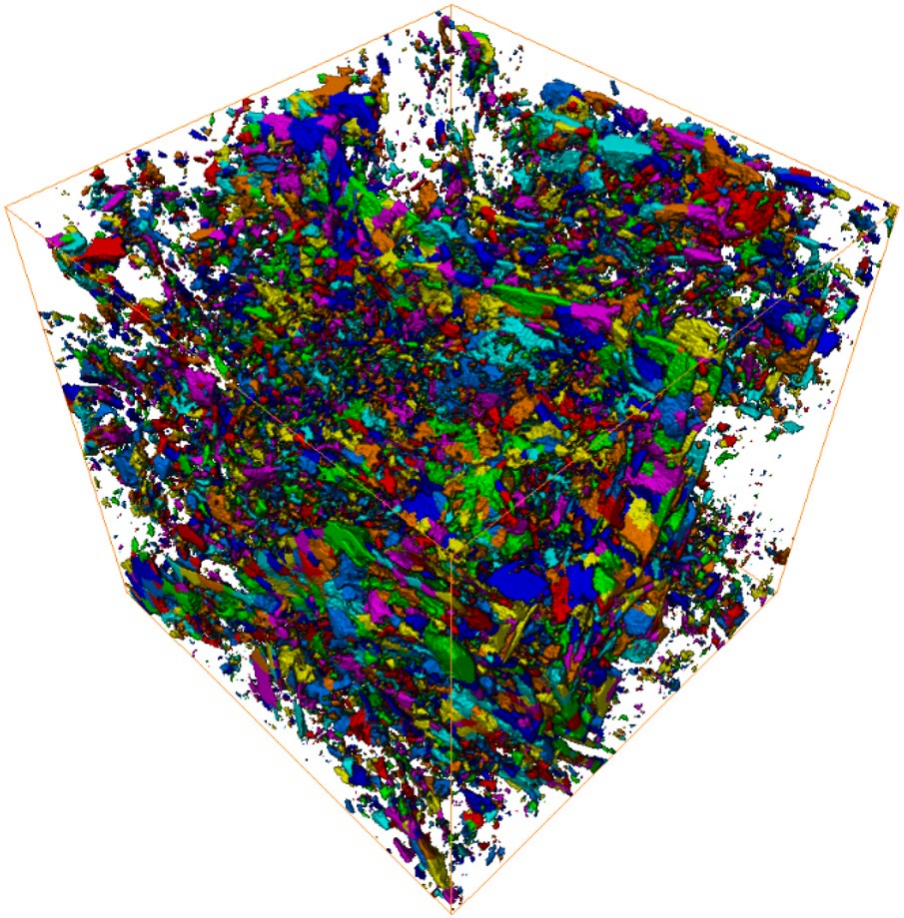
Visualization of classified pores within the pore-phase 3D model.

Figure 1 depicts the digital rock 3D model reconstructed form the processed and segmented FIB-SEM nano-tomography image dataset. The 3D model of the pore phase was then separated into particles (label field) using the distance transform and watershed-based separation algorithm, as shown in Fig. 2. This was done by calculating the particle distance map, extracting the particles skeleton (medial axis), masking the distance map with the skeleton, and then by finding and labeling the particle center (seeds) on the masked distance map.

**Figure 3:**
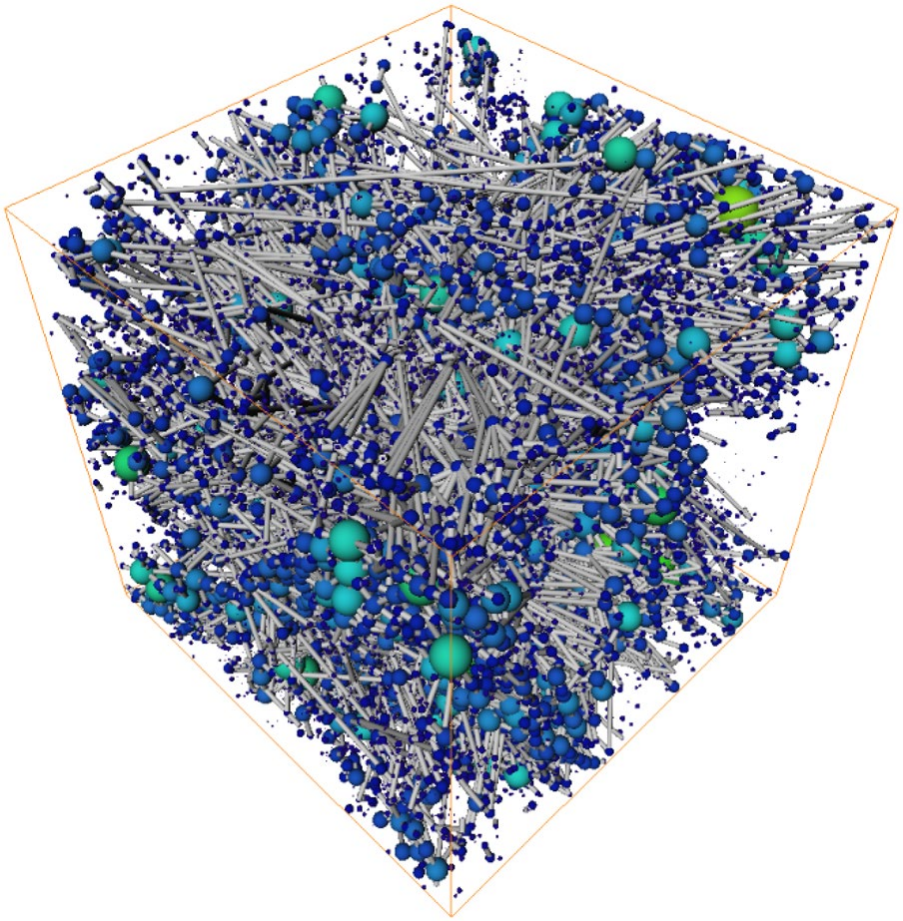
Visualization of the pore network 3D model generated from the pore-phase 3D model, in which pores are represented as a lattice of spheres connected by narrow cylinders—pore throats. The size of the pore or pore throat indicates the inscribed radius.

The separated label field was then used for pore network modeling in which pores and pore throats were extracted, as shown in Fig. 3. A pore network model (PNM) is a spatial graph in which branching or endpoints of the PNM are called pores, whereas the lines connecting these pores are called pore throats.

**Figure 4:**
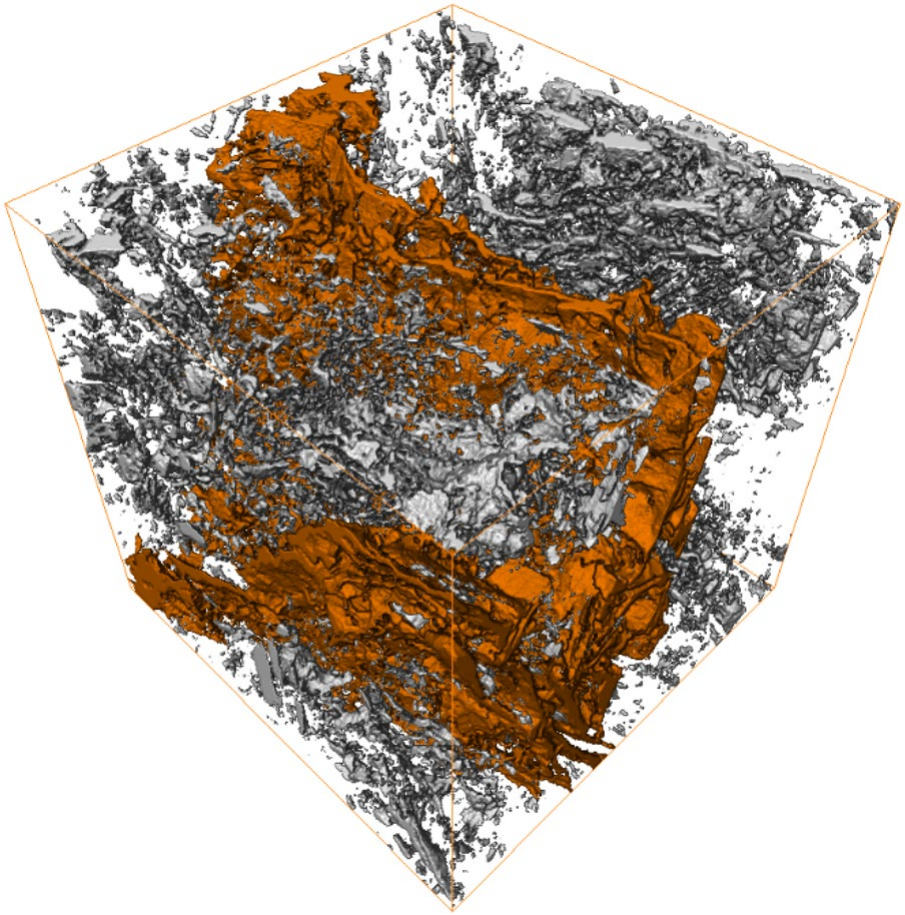
Connected (effective) porosity 3D model (orange) within the total porosity 3D model.

Next, the same 3D model of the pore phase was used for inter-connectivity analysis, in which connected (effective) and non-connected (isolated) porosities were identified, as shown in Fig. 4. The effective porosity and isolated porosity were equal to 4.5% and 2.7% respectively. The summation of both of these porosities gives the total porosity, which was equal to 7.2%. The effective pore system of a digital rock 3D model represents the pores that are responsible for fluid flow within the model, since they are inter-connected with each other to provide permeable flow pathways for e.g., hydrocarbons to move through the 3D model.

**Figure 5:**
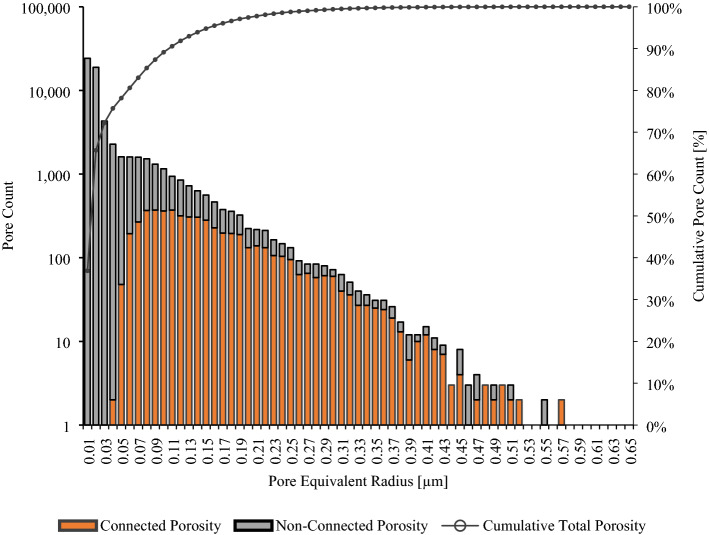
Pore size distribution of total (connected and non-connected) porosity.

Figure 5 depicts a chart with pore size distribution (PSD) comparing effective and isolated porosity within the investigated Marcellus Shale rock sample. As shown in the graph, the 3D model contained pores with pore equivalent radius that varied from approximately 10 nm to 0.6 µm, where about 80% of these pores were smaller than approximately 50 nm in diameter (as shown by cumulative pore count (of the total porosity) curve in the graph). Also, when PSDs of the effective and isolated porosity 3D models are compared, it can be observed that the vast majority of these pores were poorly connected. Only a small fraction of all the pores contributed to the effective porosity (orange bins).

### Nano-3D-printing

**Figure 6:**
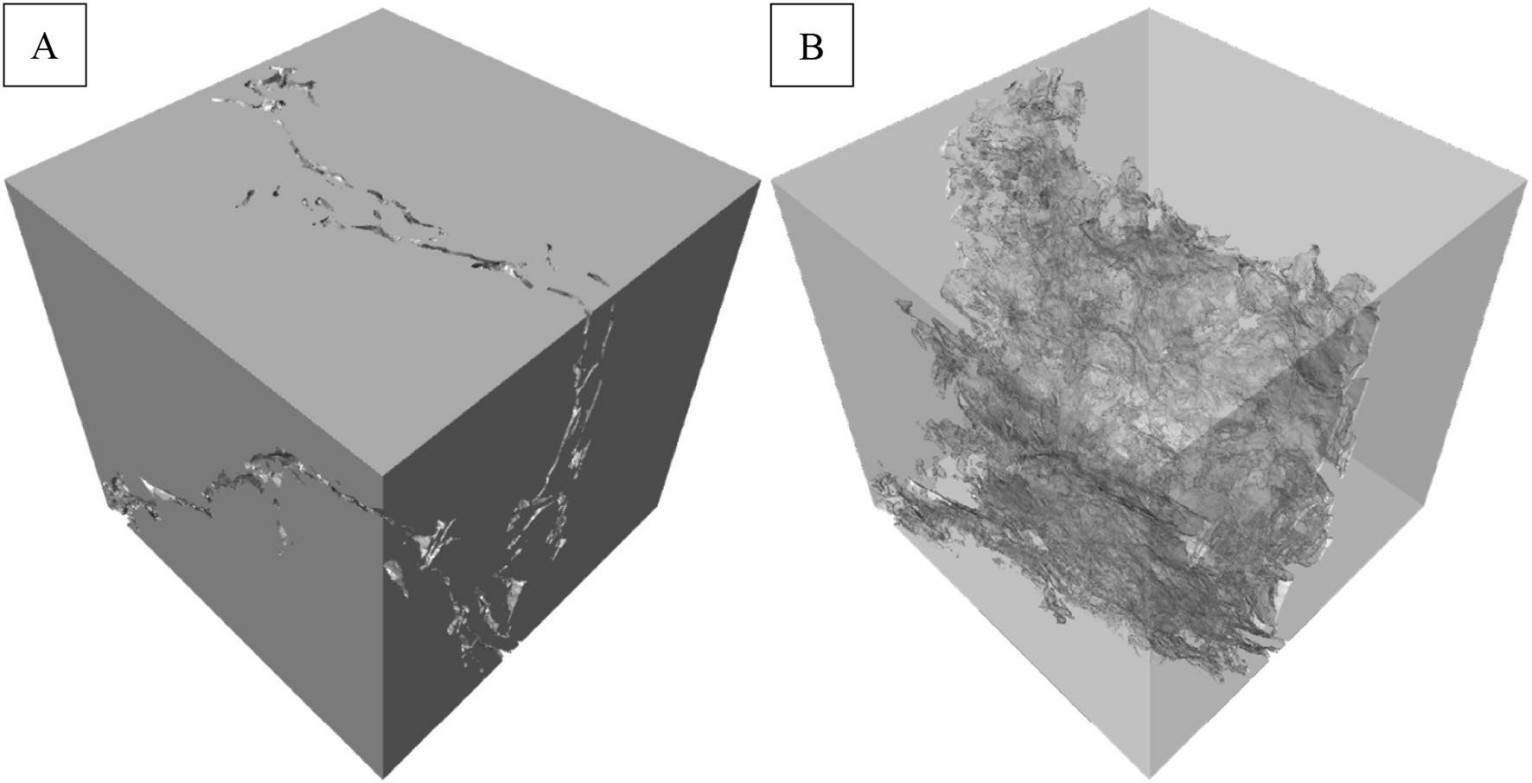
(**A**) External and (**B**) internal surface mesh of the inverted and up-scaled (10×) 3D model of the connected porosity.

The 3D model of the connected porosity was then inverted, up-scaled ten times, and used for discretization in which a simplified surface mesh was generated, as shown in Fig. 6. The surface mesh was then exported as a STL file for nano-3D-printing. The 3D model was up-scaled (from 15 × 15 × 15 µm^3^ to 150 × 150 × 150 µm^3^) because the smallest feature that can be nano-3D-printed is limited to 160 nm.

Next, the STL file was imported into the DeScribe software, which displays a preview of the 3D model and sets writing parameters, such as layer spacing or print speed. This 3D model (along with all the writing parameters) is then transferred into the NanoWrite software, which executes the print job. The nano-3D-printing—a maskless lithography nanofabrication process—is described below.

**Figure. 7:**
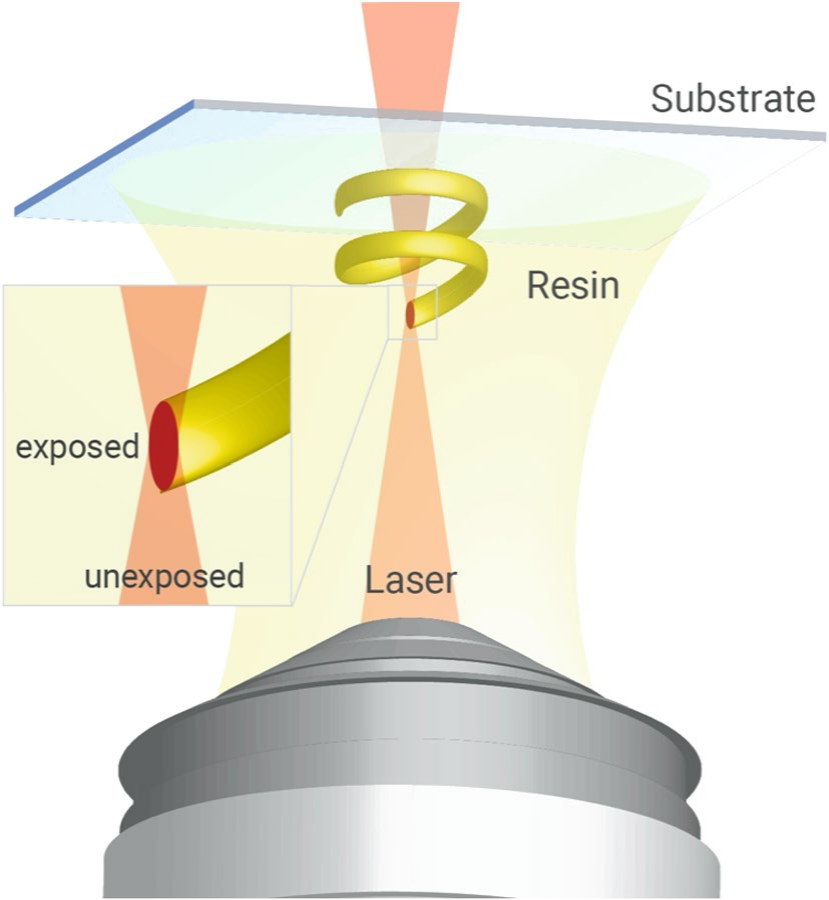
Schematic diagram illustrating the principles of operation of a nano-3D-printer. Reprinted with permission from Nanoscribe GmbH (https://www.nanoscribe.com).

**Figure 8:**
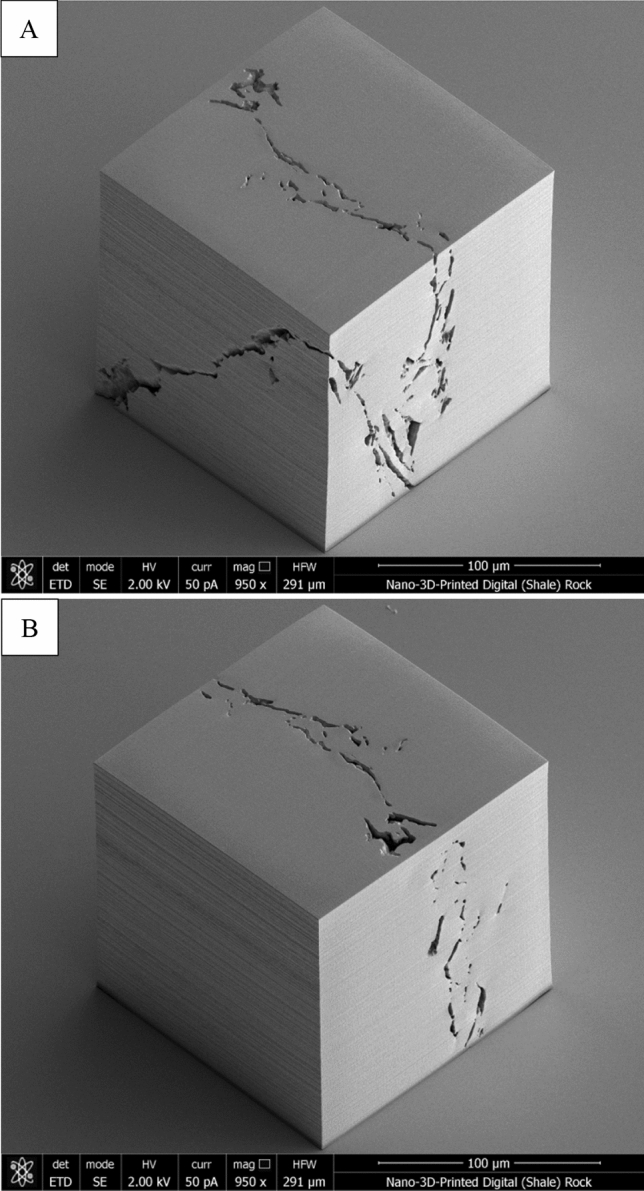
Scanning electron microscopy (SEM) image of the (**A**) front and (**B**) back of the 150 µm × 150 µm × 150 µm nano-3D-printed digital (shale) rock 3D model.

A pre-washed (with acetone, IPA, and water) substrate (piece of a silicon wafer) was fixed to a holder, and a light-sensitive polymer (photoresin) was applied. Next, the fixture was installed in a Nanoscribe Photonic Professional GT2 3D printer, which utilizes two-photon polymerization to nano-3D-print a structure. A recent review paper by Fischer and Wagener^46^ explains in-detail the principles of this process. Before nano-3D-printing, a ZEISS 63× objective immerses into the photoresin and finds the interface of the substrate using refractive index, as shown in the Fig. 7. The structure is then nano-3D-printed by scanning the laser focus in the field of view of the objective and switching the laser on and off at designated positions. By moving the objective away from the substrate (in Z-direction) and by moving the stage in X- and Y-direction, the subsequent layers build up to create the 3D model. Once the nano-3D-printing is completed, the substrate is removed and immersed in SU-8 developer (organic solvent solution). Any unexposed polymer develops away leaving the structure. Since the connected porosity 3D model was used for nano-3D-printing, the organic solvent solution should reach the entire surface area of the 3D model. The substrate/structure was then rinsed in IPA and gently dried using nitrogen. It can be followed with a UV cure if needed. The SEM image, in Fig. 8, depicts the first nano-3D-printed digital rock 3D model. Note that although the 3D model has been inspected with a SEM, which did not show any imperfections at the surface of the 3D model, the future nanofabricated structures would benefit from additional examination in 3D by nano-CT to verify that its internal structure has been properly developed.

The workflow presented in this study has been proven to be an effective way of nano-3D-printing of realistic 3D models of connected pore systems (responsible for fluid flow in shales and hence their permeability) and should be incorporated into fabrication process of microfluidics devices—(rock) lab on a chip—to study fluid flow and transport phenomena in shales.

## Summary and future work

**Figure 9:**
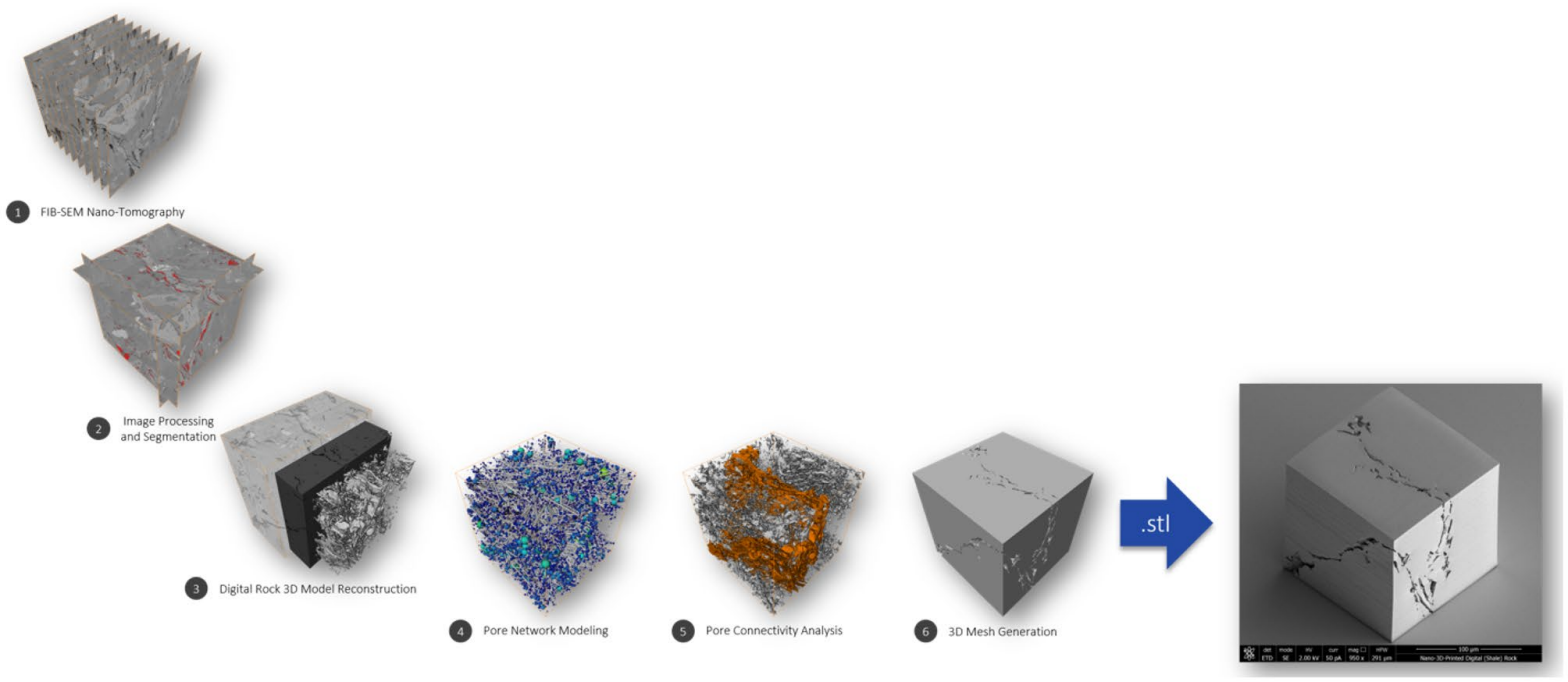
Summary of a workflow that allows to reconstruct pore network 3D models (suitable for nano-3D-printing) from processed and segmented image datasets collected using e.g., nanoscale-resolution FIB-SEM nano-tomography.

The nanoscopic features of shales—complex and heterogeneous pore systems—have been studied using a variety of sophisticated imaging techniques. It is believed that fluid behavior is markedly different in such pore networks. Perfectly calibrated materials, that mimic the pore structure of shales, are necessary for understanding this behavior. Digital rock technologies and nano-3D-printing have allowed creation of synthetic rocks. 3D-printing is revolutionizing rapid prototyping and manufacturing. Significant advances in the technology have led to creation of ‘as-designed’ objects at ultra-high resolution. Recreation of complex porous materials (often, geo-based) is another application that benefits from these 3D-printing technological advances. Creation of a synthetic nanoporous geomaterial (rock) is an involved multi-step process, which includes: advanced imaging and image analysis; pore network modeling; and nano-3D-printing of the extracted essential features of the rock using two-photon polymerization. A workflow for nano-3D-printing of digital (shale) rock 3D model, reconstructed from nanoscale-resolution 3D imaging data, has been presented for the first time. Figure 9 shows the entire workflow, from deconstruction to recreation. In this study, a 15 µm × 15 µm × 15 µm digital rock 3D model of the Marcellus Shale was reconstructed from processed and segmented FIB-SEM nano-tomography image dataset collected at 10 nm/voxel resolution. Five phases of pore space, organic matter, and mineral matter (including silicate, carbonate, and sulfide) were identified and quantified within the 3D model. The pore space 3D model was then used for pore network modeling in which pores and pore throats were characterized. The connected (effective) pore network was then converted into a surface mesh and exported as a STL file for nano-3D-printing. The digital (shale) rock 3D model was nanofabricated using two-photon polymerization-based 3D-printer and inspected with a SEM. SEM images showed no imperfections, partially validating the approach. To confirm that the desired pore network was actually printed at the expected resolution, additional imaging studies of the sample are needed. These studies are planned as more samples are printed using this workflow. Creating a microfluidic device—(rock) lab on a chip—from the printed object will also require further significant advancements. The (rock) lab on a chip will need to withstand pressure drops of several megapascals (MPa) even at very low flow rates, since flow is through nano-channels with permeabilities in the range of 10^−20^ m^2^. Creation of such devices with customized bonding schemes and study of flow versus pressure in these devices are also planned.

## Advanced imaging and image analysis methods

### Nanoscale-resolution 3D imaging with focused ion beam–scanning electron microscopy (FIB-SEM) nano-tomography

**Figure 10:**
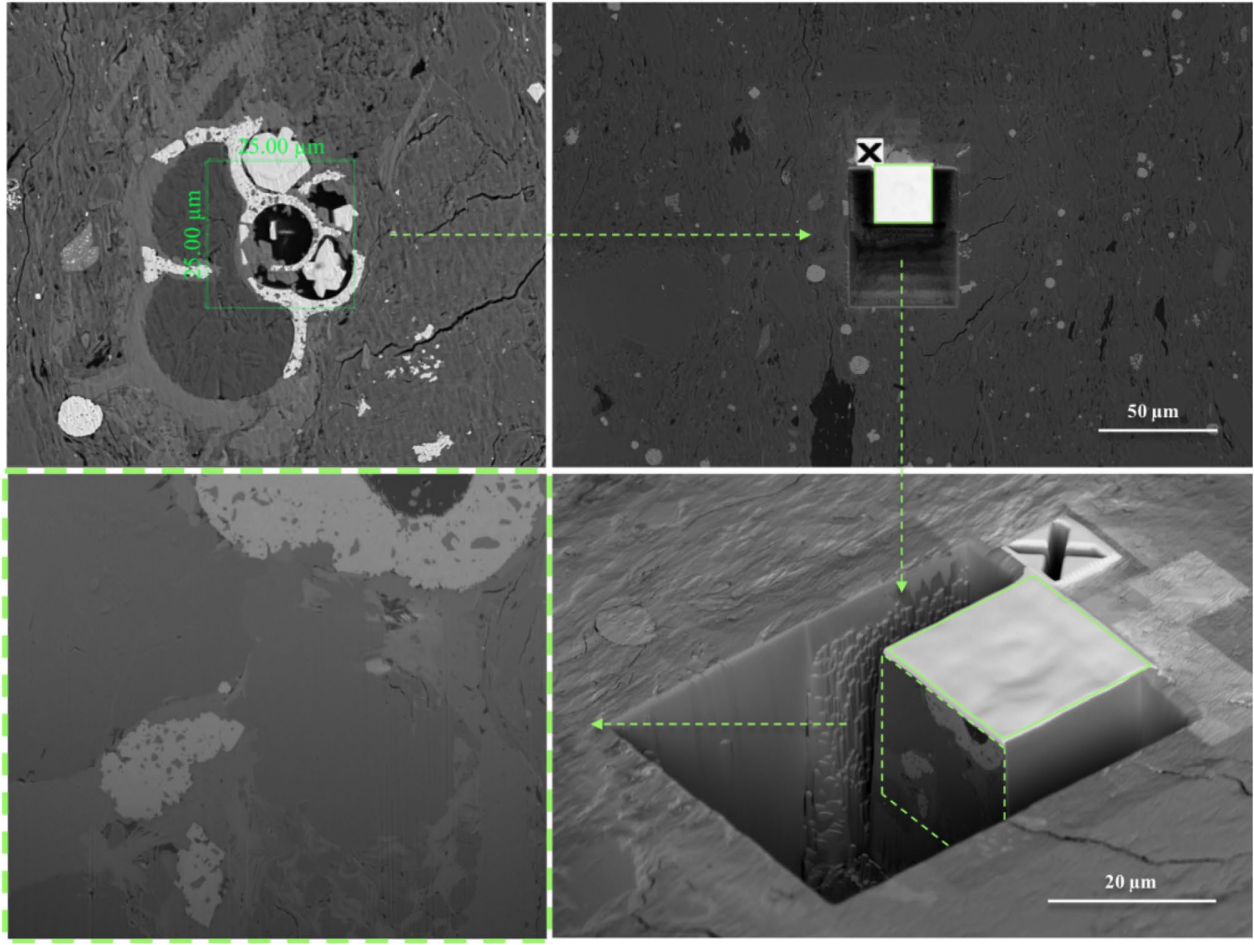
Schematic diagram illustrating an example of dual-beam focused ion beam—scanning electron microscopy (FIB-SEM) nano-tomography (serial-sectioning) experimental procedure.

FIB-SEM nano-tomography (serial-sectioning) is a nanoscale-resolution 3D imaging technique in which cross-section ion milling is used to controllably remove typically 5- to 20-nm-thin layer of material (“slice”) of the sample, and electron imaging is used to characterize the freshly prepared sample surface, as shown in Fig. 10. Automated sequential FIB milling (at 90° angle relative to the sample’s surface) and SEM imaging (at 52° angle relative to the sample’s surface) allows for the acquisition of a series of images, which in turn leads to digital rock 3D model reconstruction^47,48^.

In this study, a 15 × 15 × 15 um^3^ volume was imaged with a ZEISS Crossbeam 540 FIB-SEM at 10 nm/voxel resolution using secondary electron (SE) and backscatter electron (BSE) multi-mode at 1.5 kV acceleration voltage. The SE and BSE signals were blended into a single image to optimize contrast across pore, organic, and non-organic (mineral) phases. Approximately 1500 serial images (“slices”), with 10 nm/pixel resolution, were collected in a single-batch acquisition during automated FIB-SEM serial-sectioning (“slice-and-view”) experiment. The resulting 3D image stack was comprised of approximately 1500 × 1500 × 1500 voxels with a voxel size of 10 nm. Note, that in this paper a FIB-SEM nano-tomography image dataset was used for the workflow development, however any other nanoscale-resolution tomography image dataset (e.g., micro-/nano-CT) of a larger volume could be used. Larger-scale models may be printed with other available 3D printers. The novelty of this work is to be able to 3D-print at the resolution described using the two-photon polymerization process.

### Image analysis

The FIB-SEM nano-tomography image dataset was then processed and segmented using the PerGeos software (https://www.thermofisher.com/us/en/home/industrial/electron-microscopy/electron-microscopy-instruments-workflow-solutions/3d-visualization-analysis-software/pergeos-digital-rock-analysis.html) - in this paper, all 3D models (shown in Figs. 1, 2, 3, 4, 6 and Table 1) were generated using this software. First, during image processing, the images were re-aligned with each other, and a combination of different morphological filters (e.g., FFT, local or non-local means image processing algorithms) was applied to remove FIB-SEM imaging artifacts (e.g., curtain or shadow effect), noise, and other background intensity variations from the images. This step is essential to clean the images for easier segmentation and more accurate quantitative image analysis^49^. Second, during image segmentation, the processed FIB-SEM serial-sectioning image dataset was segmented into segments representing different phases—groups of features of the rock microstructure. Phases such as pores, organic matter, silicate, carbonate, and sulfide minerals were segmented and quantified, as shown in Table 1. Marker-based watershed (MBW) segmentation was used in which the boundaries between features are defined by the gradient function (local deviation) as the delimiter between the different features^50^. The gradient (contrast) of a SE/BSE SEM image, of different minerals of different mean atomic number, is a good indicator where the boundaries exist. The phases to be segmented were first marked with contract-based thresholding, and second, the MBW algorithm was used to compute the expansion of the markers on a landscape function, which is defined by the gradient contour. A 2D rather than 3D watershed transform is preferred in the case of FIB-SEM image datasets, since the spacing between slices is generally much bigger than the pixel size. The difference between two consecutive slices creates a high gradient in the slicing direction, blocking the usage of a 3D watershed transform.

**Table 1:** Visualization of segmented pore, organic, and non-organic (mineral) phases of the digital rock 3D model.

Phase	Volume fraction (%)	Visualization
Pores	7.2	
Organic matter	3.8	
Carbonate	8.2	
Silicate	80.7	
Sulfide	0.1	

Note that, currently, there are a number of different image processing/segmentation techniques available, including e.g., machine-learning or neural network-based deep learning protocols, which can be used during the image analysis step of the workflow (depending on the application and how satisfying the image processing/segmentation results are). In this study, a well-know MBW segmentation algorithm was used for the workflow demonstration.

## Data Availability

The data generated and/or analyzed during the current study are available from the corresponding author on reasonable request.
